# Case report: Endoscopic retrieval of a proximally migrated pancreatic stent using the basket-through- the-sphincterotome technique

**DOI:** 10.3389/fmed.2023.1230945

**Published:** 2023-08-23

**Authors:** Hang Yi, Qin Liu, Song He, Li Zhong, Xiaodong Guo, Suhua Wu, Bo Ning

**Affiliations:** ^1^Department of Gastroenterology, The Second Affiliated Hospital of Chongqing Medical University, Chongqing, China; ^2^Department of Gastroenterology, Chongqing General Hospital, Chongqing, China

**Keywords:** pancreatic stent, migration, endoscopy, sphincterotome, retrieval

## Abstract

**Background:**

The retrieval of a proximally migrated pancreatic duct (PD) stent via endoscopic retrograde cholangiopancreatography (ERCP) is technically challenging, often requiring surgical intervention. We report a case with proximal migration of a pancreatic stent that was successfully removed by a basket-through-the-sphincterotome technique.

**Case presentation:**

A 46-year-old man with prior history of chronic pancreatitis was admitted to our hospital with 1 month history of epigastric discomfort. 9 months prior he had undergone ERCP with Endoscopic sphincterotomy (EST) and a 5 Fr × 9 cm plastic pancreatic stent placement to relieve pancreatic duct stricture and abdominal pain. Magnetic retrograde cholangiopancreatography (MRCP) done this time revealed PD dilation and a stent-shaped signal inside the PD. The subsequent endoscopic ultrasonography (EUS) verified total pancreatic stent proximal migration with no visible distal end of the stent in the papilla. ERCP was performed again with an attempt to retrieve the stent. General techniques of PD cannulation with a 0.035-in guidewire over the migrated stent and balloon extraction failed. We used a mini-basket (Endoflex Germany) to replace the guidewire, which was inserted into the PD and advanced over the proximal end of the stent through the channel of the sphincterotome. The distal end of the stent was easily caught by manipulating the tip of the sphincterotome, and the stent was then pulled out. A naso-pancreatic drainage tube was placed in the main PD, and the patient was discharged 2 days after tube withdrawal.

**Conclusion:**

This was a successful case of proximally migrated pancreatic stent retrieval using the unique idea of basket-through-the-sphincterotome technique, which has rarely been reported. The basket-through-the-sphincterotome technique provides the endoscopist another way to catch the distal end without difficulty. It can improve the success rate of proximally migrated pancreatic stent retrieval, especially the pig-tail pancreatic stent, of which the sticking of the proximal end into pancreatic duct branches often makes the distal end the only choice to retrieve.

## Background

Pancreatic duct stents are applied in many pancreatic diseases, such as benign or malignant strictures of the PD and pancreatic ductal stones, and in prophylactic use for post-ERCP pancreatitis (PEP) (1). With the increasing use of PD stents, complications are well recognized, including infection, bleeding, stent migration, ductal perforation, PD or duodenal damage, and stent migration. Proximal migration of pancreatic stents is an important complication of PD stenting and occurs with a reported incidence of approximately 5.2%, it may induce serious situations of pancreatitis and morphological injuries of the PD if left without removal, and approximately 10–17% of patients with migration have to undergo surgical interventions (2, 3).

The retrieval of proximally migrated PD stents can be technically difficult; can usually be achieved endoscopically by using forceps (4), snares (5), balloons (6), basket catheters (1), or pancreatoscopy techniques (7); and sometimes requires surgical interventions. If a pig-tail stent migrates proximally, the curled tail often sticks into the branches of the pancreatic duct. In this situation, general forceps, snares, baskets, or balloon techniques often fail. Here, we report a case of a proximally migrated PD stent that was successfully removed using a specific basket-through-the-sphincterotome technique, which easily captured the proximal end of a migrated stent by manipulating a mini-basket through the tip of the sphincterotome.

## Case presentation

**Figure 1:**
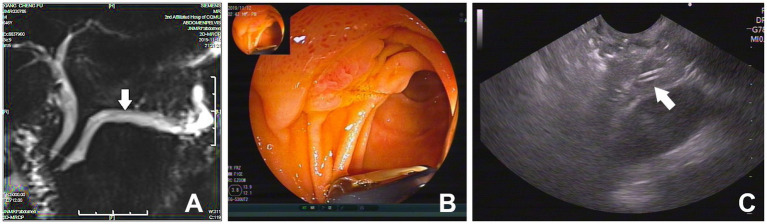
**(A)** MRCP revealed a stent-shape like signal inside the dilated PD (white arrow); **(B)** The end of the PD stent was not observed endoscopically under EUS; **(C)** EUS found a hyperechoic equal sign signal of stent in the PD (white arrow).

A 46-year-old man presented with epigastric discomfort for 1 month, and a regimen of proton pump inhibitors offered no symptomatic relief. He received ERCP 9 months prior for frequent relapses of chronic pancreatitis. During the operation, EST and pancreatic stone removal were performed, and then a 5 Fr × 9 cm straight plastic stent was placed in the PD to relieve the stricture and abdominal pain. At admission, the results of routine blood, amylase, and lipase tests and urine and stool tests were all within normal ranges, as were those of liver and kidney function tests. Magnetic retrograde cholangiopancreatography (MRCP) revealed PD dilation and a stent-shaped signal inside the PD (Figure 1A). Pancreatic stent migration was considered at the time. To confirm this, endoscopic ultrasonography (EUS) was applied. During the examination, no end of the stent was found inside the papilla under the endoscopic view. An examination of the pancreatic duct found a high-echo signal similar to that of a stent inside the main pancreatic duct. The total proximal migration of the stent into the PD was then confirmed (Figures 1B,C).

**Figure 2:**
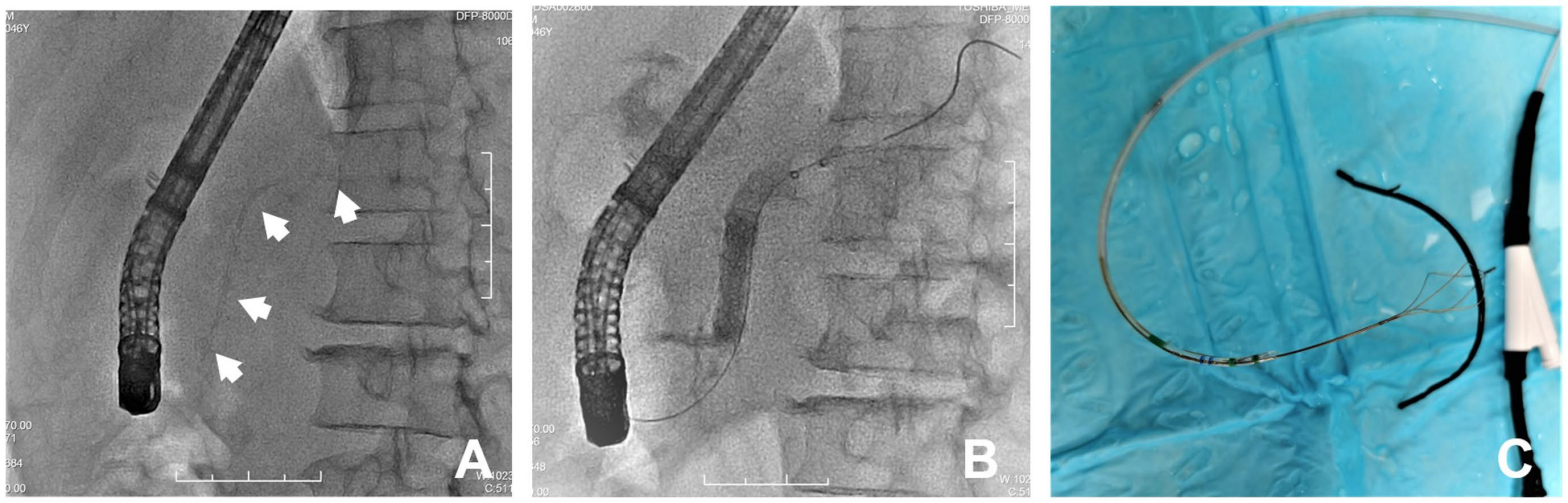
**(A)** Fluroscopy showed a proximal migrated PD stent (white arrows); **(B)** Balloon extraction of the PD stent failed; **(C)** The stent was successfully retrieved by the through-the-sphincterotome-basket technique.

Once the migration of the PD stent was confirmed, retrieval by ERCP was first considered and performed in our endoscopy center. The migrated stent was identified in the location of the body of the PD (Figure 2A). The cannulation of the PD was uneventful. A 0.035-in-wide guidewire (Boston Scientific) was then placed over the migrated stent into the tail of the PD. An 8–10 mm balloon was used in an attempt to drag the stent out. It failed (Figure 2B). Forceps along the guidewire were tried but also failed as it was too hard to pass the forceps through the stricture of the PD. A general basket was then used. It was advanced over the proximal end of the stent, and several attempts to catch the proximal end failed due to incorrect angle and direction. The adjustment of the basket was hard because of the physiological bend of the pancreatic duct. To overcome this, we put the sphincterotome over the proximal end of PD stent again and then withdrew the guidewire, which was replaced by a special mini-basket (Endoflex, Germany), through the channel of the sphincterotome. When the basket was released, it was just near the proximal end. We adjusted the basket in the right direction and position easily by bending and rotating the handle of the sphincterotome, which made it quick to grasp the proximal end and retrieve the stent (Figure 2C). After that, a naso-pancreatic drainage tube was placed in the main PD to prevent PEP. The recovery after ERCP was uneventful, and the patient was discharged 2 days later.

## Discussion and conclusion

With the wide use of pancreatic duct stents, more adverse events related to stents have become a challenge to endoscopists. The migration of PD stents is one of the most concerning events because of its difficult handling. In published articles, the reported migration rates of distal and proximal pancreatic stents were 7.5 and 5.2%, respectively, and the risk factors for proximal PD stent migration might be associated with malignant strictures, larger diameter stents, sphincter of Oddi dysfunction, and longer stents (2, 8, 9). Distal migration is less harmful as the PD stent can be excreted from the intestinal tract; comparatively, proximal migration is likely to result in pancreatitis or damage to the PD.

The management of a proximally migrated pancreatic stent is technically difficult. The small diameter and curved morphology of the pancreatic duct often give limited space for instrument retrieval. Up to 78% of proximally migrated stents can be retrieved endoscopically (10), and surgical intervention proceeds only when multiple endoscopic retrieval fails (11). The general endoscopic retrieval methods include forceps (28%), balloons (44%), and baskets and snares (11%) (13). Most of these methods are applied with the purpose of catching or trapping the migrated stents and then dragging or pulling them out. Sometimes PD dilation with balloons is needed due to PD stricture. In previous studies, the post-ERCP pancreatitis (PEP) risk was high, with difficult cannulation and multiple attempts at migrated PD stent retrieval. The increased incidence of PEP is related to relevant pancreatic mechanical trauma (8, 12). Most of the techniques were reported in sporadic case reports or some retrospective analyses. The efficiency and safety could not be evaluated by prospective studies (1, 4–8).

**Figure 3:**
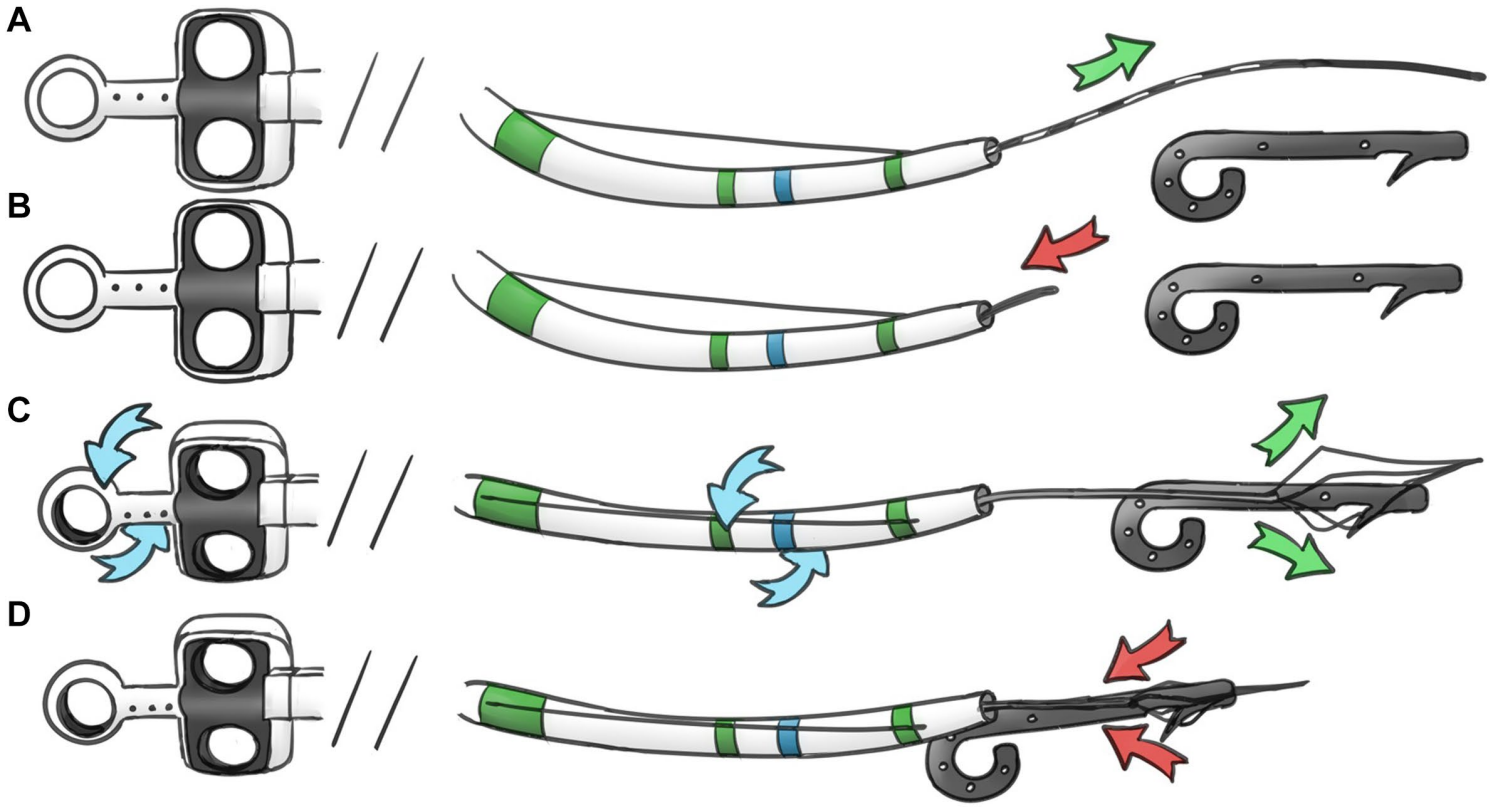
Schema showing the removal procedure. **(A)** Advancing the guidewire over the proximal aspect of migrated PD stent. **(B)** Exchange the guidewire to minimal basket. **(C)** Manipulating the sphincterotome and the basket to grasp the proximal end of stent. **(D)** Capture and retrieval of the PD stent.

**Figure 4:**
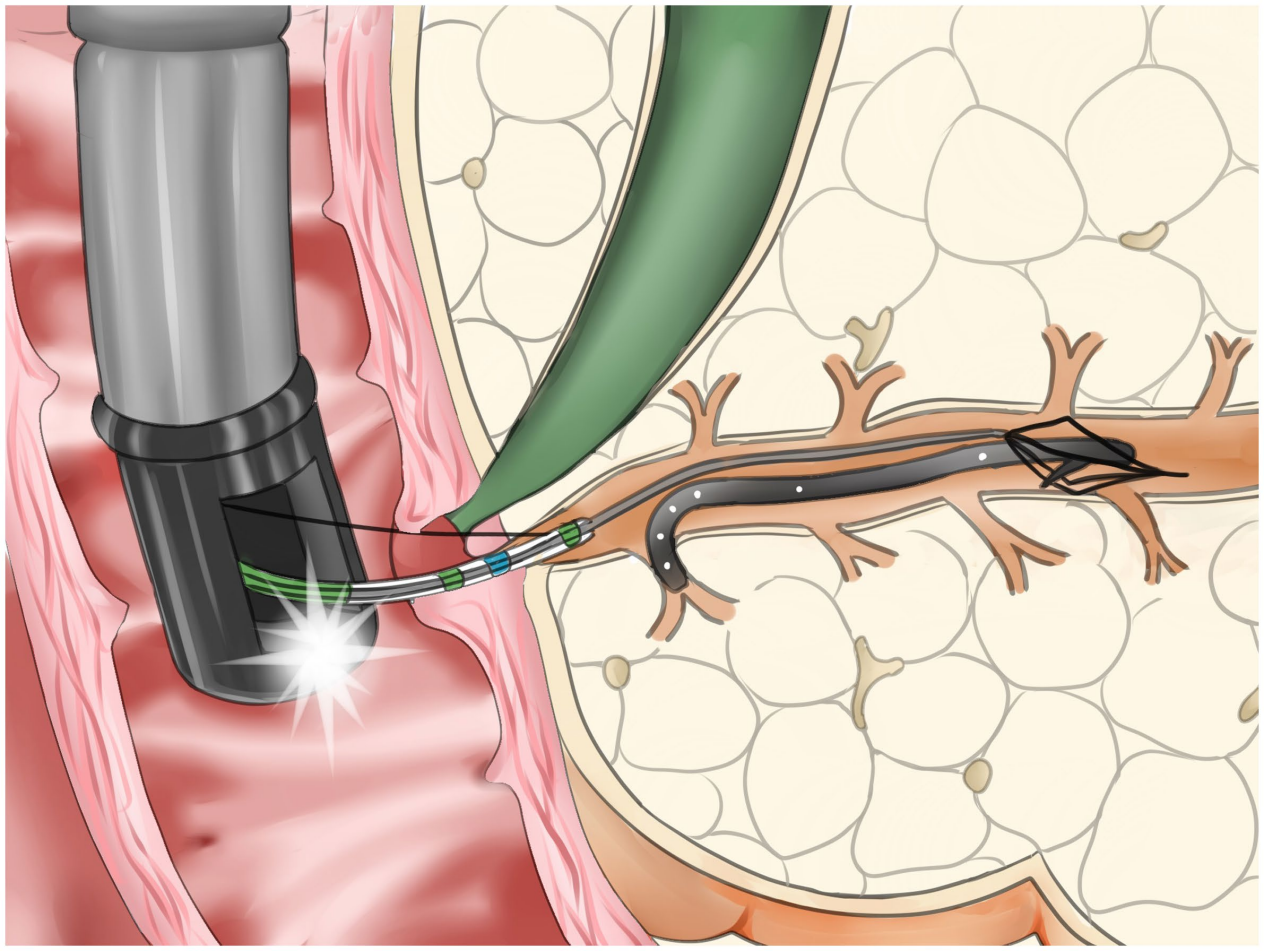
Schema showing a proximally migrated stent with curled end sticking into a branch of PD captured and retrieved with through-the-sphincterotome-basket technique.

With the development of endoscopic equipment and techniques, new methods have been applied recently. In a normal-sized PD, retrieval was performed by using one-sided opening-cup biopsy forceps with a 0.021-in-wide guidewire threaded through the hole of the opened cup (4). The stent-in-stent method is a good method of retrieval. However, the migrated stents can be handled only when the diameter is less than 5 French; otherwise, no larger stents are available to entangle them (2, 14). Pancreatoscopy is another novel technique to remove the proximally migrated PD stent; owing to the small diameter, the stent should first be dragged down from the deeper portions of the PD (7). Traditional baskets, balloons, and forceps have the potential to cause complications such as PD injury, hemorrhage, or perforation during cannulation even with the aid of guidewires. In the situation of a narrow PD combined with pancreatic duct stones, the complications and failure rate are even higher (1, 13, 15, 16). Other novel methods, such as the gooseneck snare method (5), turned guidewire looping method (17), modified snare with a cut plastic sheath method (18), wedge technique (19), lasso technique (20), and handmade catheter with a guidewire loop method (21), are effective methods for the retrieval of proximally migrated PD stents. Some of these methods require specific instruments, and the manipulations are complicated and time consuming. Compared with those techniques, the basket-through-the-sphincterotome technique uses a mini-basket that passes through the channel of the sphincterotome after successful pancreatic cannulation and guidewire withdrawal. Once the mini-basket replaces the guidewire and situates inside the sphincterotome, it can be easily manipulated upwards or downwards by bending and releasing the sphincterotome and turning to the proper direction or position by rotating the handle. It is easy and effective to grasp the distal or proximal end of the stent. This technique reduces the operating time and the number of attempts and minimizes injury to the papilla orifice and pancreatic duct (Figure 3). When pig-tail pancreatic stents migrate proximally, the curled distal end often sticks into pancreatic duct branches. In this situation, it is hard to adjust the direction and position of the general basket, snare or balloon and almost impossible to catch any end of the stent (Figure 4). By adjusting the sphincterotome, through which the mini-basket can be driven, the basket-through-the-sphincterotome technique is able to finish grasping and pulling the stent quickly and effectively, regardless of stent size. The PD diameter, natural curve, and limited space have minimal effects on this method. In conclusion, endoscopic retrieval of a proximally migrated PD stent with a mini-basket through the sphincterotome is safe and effective. It can be a good choice for the handling of difficult proximally migrated pancreatic stents.

## Data availability statement

The original contributions presented in the study are included in the article/supplementary material, further inquiries can be directed to the corresponding author.

## Ethics statement

The studies involving human participants were reviewed and approved by Ethics Committee of the Second Affiliated Hospital of Chongqing Medical University. The patients/participants provided their written informed consent to participate in this study. Written informed consent was obtained from the individual(s) for the publication of any potentially identifiable images or data included in this article.

## Author contributions

HY made substantial contributions to the case report and research work. The initial draft of the manuscript was co-written by HY, QL, SH, and BN. LZ expended much effort in acquiring clinical data and schema drawing. XG and SW were tasked with important intellectual content revisions. All authors contributed to the article and approved the submitted version.

## Funding

This work was supported by Chongqing Natural Science Foundation project (CSTB2022NSCQ-MSX0130).

## Conflict of interest

The authors declare that the research was conducted in the absence of any commercial or financial relationships that could be construed as a potential conflict of interest.

## Publisher’s note

All claims expressed in this article are solely those of the authors and do not necessarily represent those of their affiliated organizations, or those of the publisher, the editors and the reviewers. Any product that may be evaluated in this article, or claim that may be made by its manufacturer, is not guaranteed or endorsed by the publisher.

## Abbreviations

ERCP: Endoscopic retrograde cholangiopancreatography
MRCP: Magnetic retrograde cholangiopancreatography
PD: Pancreatic duct
PEP: Post-ERCP pancreatitis
EST: Endoscopic sphincterotomy
EUS: Endoscopic ultrasonography
